# Renin-Angiotensin System Gene Variants and Type 2 Diabetes Mellitus: Influence of Angiotensinogen

**DOI:** 10.1155/2016/2161376

**Published:** 2015-11-22

**Authors:** Siew Mei Joyce-Tan, Shamsul Mohd Zain, Munavvar Zubaid Abdul Sattar, Nor Azizan Abdullah

**Affiliations:** ^1^Department of Pharmacology, Faculty of Medicine, University of Malaya, 50603 Kuala Lumpur, Malaysia; ^2^School of Pharmaceutical Sciences, University Sains Malaysia, 11800 Penang, Malaysia

## Abstract

Genome-wide association studies (GWAS) have been successfully used to call for variants associated with diseases including type 2 diabetes mellitus (T2DM). However, some variants are not included in the GWAS to avoid penalty in multiple hypothetic testing. Thus, candidate gene approach is still useful even at GWAS era. This study attempted to assess whether genetic variations in the renin-angiotensin system (RAS) and their gene interactions are associated with T2DM risk. We genotyped 290 T2DM patients and 267 controls using three genes of the RAS, namely, angiotensin converting enzyme (*ACE*), angiotensinogen (*AGT*), and angiotensin II type 1 receptor (*AGTR1*). There were significant differences in allele frequencies between cases and controls for *AGT* variants (*P* = 0.05) but not for *ACE* and *AGTR1*. Haplotype TCG of the *AGT* was associated with increased risk of T2DM (OR 1.92, 95% CI 1.15–3.20, permuted *P* = 0.012); however, no evidence of significant gene-gene interactions was seen. Nonetheless, our analysis revealed that the associations of the *AGT* variants with T2DM were independently associated. Thus, this study suggests that genetic variants of the RAS can modestly influence the T2DM risk.

## 1. Introduction

Type 2 diabetes mellitus (T2DM) is a major public health concern and affected patients stand a higher risk of suffering the injurious effects of hyperglycemia such as coronary artery disease, peripheral arterial disease, and ultimately stroke [1]. Prolonged hyperglycemia could lead to the development of microvascular complications such as diabetic nephropathy, neuropathy, and retinopathy [2]. The number of individuals with diabetes is on the rise and the figure is expected to reach 552 million by 2030 owing to many factors such as population growth, aging, urbanisation, obesity, and physical inactivity [3]. Although environmental factors are reasons often considered, it is important to keep in mind that environmental factors, by themselves, represent only a fraction of cases. Individuals with family history of T2DM are at higher risk of developing the disease as shown in familial studies [4] suggesting strong genetic contribution.

Now that the genome-wide association studies (GWAS) are feasible, allowing genetic data to be collected at unprecedented rates, many disease-associated alleles have been identified through GWAS and applied to T2DM [5]. Notwithstanding that, one cannot deny that the candidate gene approach has been a pioneer and at a forefront of genetic association studies. The so-called genome-wide significance level (*P* < 5 × 10^8^) has limited the common and rare variants capturing and hence contribute to the missing heritability. Thus, candidate gene approach is still valuable even at the GWAS era. The roles of renin-angiotensin system (RAS) in insulin signaling pathway and insulin resistance have been well documented [6]. The blockade of the system has been shown to have beneficial effects in the prevention of T2DM [7]. These findings strongly imply that the variations in RAS might be associated with the onset of T2DM. Nevertheless, the underlying genetic mechanisms of the RAS and susceptibility to T2DM remain poorly understood.

In this study, we examined the association of genetic variations of the RAS with susceptibility to T2DM in the Malaysian Malays. While most of the reports are centered on the Western populations, little is known about the data from the Asians. Furthermore, the Malaysian Malays differ in terms of population structure among other Asians including the Chinese and Japanese [8]. However, whether or not the genetic variations of the RAS contribute to the risk difference of T2DM remains to be investigated and is a hypothesis of this study. We also examined the gene-gene interactions within the system in order to understand the gene-gene effect on the occurrence of T2DM.

## 2. Methods

### 2.1. Study Participants

This study consisted of 557 Malay participants, 290 of which are T2DM patients and 267 are controls recruited from the University Malaya Medical Centre (UMMC). T2DM patients and controls were matched by age with mean age of 57.4 and 57.0, respectively. T2DM was diagnosed by qualified clinicians based on fasting blood glucose (FBG) levels ≥7.0 mmol/L for two consecutive routine screen readings and glycated haemoglobin (HbA1c) ≥6.5% (≥48 mmol/mol) with normoalbuminuria, and without a history of any renal complications. Controls were those coming for routine health screening at clinics, with no evidence or family history of T2DM. The study was approved by the medical ethics committee of the UMMC and was performed according to the Declaration of Helsinki. Written informed consent was obtained from all study participants prior to participation into the study.

### 2.2. Laboratory and Clinical Data

Anthropometric data such as height (cm), weight (kg), body mass index (BMI, kg/m^2^), and waist circumference were determined as standard protocol. Measurement of blood pressure (mmHg) was according to standard recommendation and clinical practice guidelines. The biochemical tests for the determination of fasting blood glucose (mmol/L), HbA1C (%, mmol/mol), serum urea (mmol/L), serum creatinine (mmol/L), glomerular filtration rate (GFR) (mL/min/1.72 m^2^), serum triglyceride (mmol/L), serum cholesterol (mmol/L), high density lipoprotein (HDL) (mmol/L), and low density lipoprotein (LDL) (mmol/L) were according to standard clinical laboratory methods carried out in an accredited laboratory at UMMC. Other clinical data included were information on duration of diabetes, presence of any complication, and history of other disorders.

### 2.3. Genotyping

Genomic DNA was extracted from peripheral blood leucocytes using the QIAamp DNA Blood Mini Kit (Qiagen, Valencia, CA, USA) according to manufacturer's protocols. Genotyping was carried out for the selected variants: angiotensin converting enzyme (*ACE*) rs4344, rs4359, rs4363, rs4459609, rs1800764, and* ACE Insertion/Deletion*; angiotensinogen (*AGT*) rs699, rs4762, and rs5051; and angiotensin II type 1 receptor (*AGTR1*) rs388915. Amplification and quantitative real-time RT-PCR analyses were carried out by using Step One Real Time PCR system (Applied Biosystems, USA). The total reaction volume for each well was 10 *μ*L containing 5 *μ*L (2x) TaqMan Genotyping Master Mix (Applied Biosystems, USA), 0.5 *μ*L customized assay mix (20x) containing sense and antisense primers and TaqMan probe (Applied Biosystems, USA) specific to each gene, 3.5 *μ*L distilled water, and 1 *μ*L (10–20 ng) genomic DNA. Cycles were 60°C for 30 sec, a 10 min 95°C denaturing step, followed by 40 cycles of 95°C denaturation, incubated at 60°C for 1 min, and denatured at 95°C for 15 sec for the final step.

### 2.4. Statistical Analysis

All statistical tests were performed using SPSS version 18.0 (IBM Corp., Chicago, IL, USA), otherwise mentioned. Data were presented as percentage or mean ± standard deviation (S.D.). Categorical and continuous variables were compared between patients with diabetes and patients without diabetes using Pearson's *χ* ^2^ test, independent *t*-test, and Mann-Whitney *U* test as appropriate. Hardy-Weinberg equilibrium (HWE) was checked for the genotype distribution prior to genetic analysis using a goodness of fit *χ* ^2^ test. Deviation from HWE was called when the *P* < 0.05. Odds ratios and 95% confidence interval (CI) for the findings were computed using logistic regression. Multivariate analysis revealed that gender, waist circumference, and waist-to-hip ratio (WHR) are contributing factors associated with T2DM. Gender was collapsed into men and women in the subsequent analysis; thus, adjustment would render unnecessary. Waist circumference was used in the multiple logistic regression as suggested earlier [9]. Correction for multiple testing was performed using Bonferroni's method. Linkage disequilibrium (LD) and haplotype analyses were computed using Haploview 4.2 program. *P* values for haplotype analysis were calculated based on 100,000 permutations. The odds ratio for the haplotype association was calculated using R program version 2.11.1. Parameter comparisons among genotypes were tested using Analysis of Variance (ANOVA) and Kruskal-Wallis as appropriate. Linear regression was used to assess the correlation between genetic variants and clinical parameters for normally distributed variables; otherwise, Spearman's correlation test was adopted. A two-sided *P* value of <0.05 is considered to be statistically significant. To investigate the influence of gene-gene interaction on T2DM, Generalized Multifactor Dimensionality Reduction (GMDR) method was employed. All possible interactions were tested using 10-fold cross validation with exhaustive search, which considers all possible variable combinations.

## 3. Results

The demographic and clinical data of the participants are shown in Table 1. Gender was matched between patients and controls. Patients and controls were significantly differed in waist circumference, WHR, fasting blood glucose, HbA1c, serum urea, serum creatinine, serum triglyceride, serum cholesterol, high density lipoprotein, and low density lipoprotein (*P* < 0.05). Knowing gender as a risk for diabetes and Asian men are particularly at more risk for the disease [10], we further described the comparison in male. Significant differences were also observed in waist circumference, WHR, fasting blood glucose, HbA1c, serum creatinine, serum cholesterol, and low density lipoprotein in men with diabetes and controls (Table 2).

### 3.1. RAS Variants and T2DM

Assessment of genotype distributions in both T2DM patients and controls indicated no deviation from HWE, thus, providing confidence for the genetic analysis. None of the investigated genetic variants were associated with T2DM in the overall participants as summarised in Supplementary Table 1 in Supplementary Material available online at http://dx.doi.org/10.1155/2016/2161376. Current opinion from the experts has suggested that men are biologically more susceptible and develop diabetes at even lower BMI than women [11]. Taking this into account, we hypothesize that gender is a contributing factor and may affect the study results. Surprisingly, analysis by gender revealed that the genetic variants of* AGT* were significantly associated with T2DM only in male (Table 3). However, none of the genetic variants within* ACE* and* AGTR1* were associated with T2DM (Supplementary Table 2).

The* AGT* rs699-C and rs4762-T alleles were significantly higher in the controls compared to the patients (adjusted OR 0.47, 95% CI 0.27–0.81, *P* = 0.007 and adjusted OR 0.54, 95% CI 0.29–0.98, *P* = 0.042, resp.), suggesting that these variants are associated with reduced risk of T2DM. Nonetheless, the* AGT* rs5051-G allele was associated with 1.94-fold increased risk of T2DM (*P* = 0.017). These findings, after adjustments for the confounding factors, clearly indicated that the variants were independent predictors for T2DM. Although our initial multivariate analysis found no contributing effect of age and BMI, we ought to include these variables during the adjustment as they are strong predictors for the development of T2DM [12]. Differences observed between the patients and controls, especially in fasting blood glucose and HbA1c, were not adjusted for as these are diabetes-associated risk factors. While dyslipidemia was excluded as it is attributed to metabolic disorder from diabetes. Following correction for multiple testing (0.05/6), only* AGT* rs699 *P* value remained significant.

### 3.2. Linkage Disequilibrium and Haplotype Association of* AGT* Variants in the Male Gender

*AGT* variants appeared to be in strong (*D*′ > 0.98) linkage disequilibrium (LD), suggesting the tendency of the single nucleotide polymorphisms (SNPs) to be coinherited. A small set of tag SNP would therefore be sufficient to capture the genetic information of the gene. In this study, three haplotypes inferred from rs699, rs4762, and rs5051 (CCA, TCG, and CTA) were yielded with frequencies above 5% (Table 4). Haplotype CCA (71%) was the most frequent followed by TCG (16%) and CTA (13%). Haplotype analysis revealed that TCG was found to be associated with T2DM (OR 1.92, 95% CI 1.15–3.20, permuted *P* = 0.012). Tag SNP was reported for* AGT *rs699.

### 3.3. RAS Gene-Gene Interactions

In order to understand the genetic basis of T2DM, we evaluated the interactions at the gene level. Analysis however indicated a lack of evidence for gene-gene interactions within the RAS.

### 3.4. *AGT* rs699 Variant and Association with Clinical Parameters

We chose to explore the relationship between the* AGT* rs699 and clinical parameters for the following reasons, (i) the evidence of strong *P* value in single marker analysis and (ii) rs699 being a tagging SNP. There was no difference in the levels of clinical parameters among the genotypes except for FBG and HbA1c (*P* = 0.005 and *P* = 0.012) (Supplementary Table 3). These observations strongly imply that the observed genetic association findings are likely to hold true and intriguingly; the differences were also seen in men (*P* = 0.005 and *P* = 0.01) but not in women. We then sought to investigate whether the variant was also correlated with the levels of the parameters. We demonstrated marginal negative correlations with FBG and HbA1c (*P* = 0.06 and *P* = 0.052); associations however improved when analysed in men (*P* = 0.04 and *P* = 0.017). The results suggest that* AGT *variants were independently associated with T2DM.

## 4. Discussion

Genome-wide association study (GWAS) is a comprehensive and unbiased approach that is able to identify candidate gene markers encompassing the entire genome [13]. However, a major stumbling block to the successful execution of the GWAS is the high cost for exhaustive genotyping due to enormous number of SNPs in the entire genome. The efficiency of GWAS often rests on a set of tag SNPs that serve as proxies for the uncollected SNPs. Together with the fact that frequency of allele is often population specific, there may still be a need for candidate gene approach. In this study, we examined the association of genetic variants of the RAS with T2DM risk. Some of the variants are reported for the first time in the present study. We also examined whether there are gene-gene interactions within the RAS that can influence patient's susceptibility to T2DM. We showed that allele frequencies of the* AGT* variants were significantly different between the T2DM patients and controls, suggesting genetic predisposition to T2DM.

Among the studied genes, only* AGT* was shown to be associated with T2DM and this association was seen only in men. Two* AGT* variants rs699 and rs4762 were associated with reduced risk of T2DM while rs5051 was associated with increased risk. Intriguingly, these three variants displayed similar linkage disequilibrium structure and the haplotype TCG was associated with increased risk of T2DM. Risk of diabetic nephropathy was previously shown to be associated with enrichment of this haplotype in the Tunisians [14]. Nevertheless, exhaustive search of the literatures failed to find any study that examined the haplotype association with T2DM, making the present study the first to report this haplotype association.* AGT* rs699-C allele was previously found to be associated with increased risk of T2DM in the Pakistani [15], despite no significant association in the Chinese [16] and recently the Japanese [17]. The Pakistani and the Japanese were not investigated by gender comparison whilst the Chinese demonstrated neither association in men nor women. Although these published studies and the present study shared a common study population which is of Asian descendent, the direction of findings was however different among studies, indicating that population-specific candidate gene study is still useful. Pakistan is one of the Asian countries reported with high prevalence of diabetes compared to the Malay, Chinese, and Japanese [18]. To date, there is only one report on the association between* AGT* rs4762 and diabetes [19]. The study examined the association of* AGT* rs4762 with diabetes in a post kidney transplant patients in Korea and found that* AGT* rs4762 is associated with increased risk of diabetes. Interestingly, the direction of finding from the Korean also differs from us, suggesting ethnicity differences may contribute to the conflicting results. It should also be noted that the Korean study did not compare the gender. As for* AGT* rs5051, to the best of our knowledge, there is no published report on the association with diabetes, and our study provides a novel report. We also failed to demonstrate any significant association between variants within the* ACE* and* AGTR1* variants.

Genetic association studies between the RAS and T2DM have not been extensively studied. Several RAS-related genes interactions including* ACE* Ins/Del were reported in the Chinese T2DM patients, but these associations were not seen in single locus marker except for* ACE* Ins/Del in women [16]. Nonetheless, interactions between the RAS genes were not shown in our study. A meta-analysis conducted in different Chinese ethnics revealed no significant association between* ACE* Ins/Del and T2DM [20], which is supported by a Japanese study [17]. Together, these observations indicate that there are conflicting results in a small set of studies, thus pointing for the need for further investigation into this relationship. In this study,* AGT* gene showed a modest but significant influence toward the outcome of T2DM. The mechanisms of the* AGT* variants in causing T2DM are currently unclear, but it is likely due to the impact of angiotensinogen levels. Angiotensinogen is an adipokine component of the RAS that is involved in the earlier part of the system cascade. The angiotensinogen mRNA levels are highly expressed in the visceral rather than the subcutaneous adipose tissue region [21]. Despite presenting with no biological activity, once broken down to angiotensin II (AngII) via a consecutive action of renin and ACE, it can induce insulin resistance via a cross-talk mechanism between insulin and AngII signaling cascade [6]. Transitional changes of amino acid methionine to threonine (rs699) and threonine to methionine (rs4762) in the exonic regions of AGT lead to alterations of the protein function thereby regulating the* AGT* expression and subsequently plasma angiotensinogen levels [22]. Carriers of rs699-C allele are reported with higher serum angiotensinogen levels [23]. On the other hand, the* AGT* promoter variant rs5051 has been associated with an* in vitro* increase in* AGT* expression [24].

In this study, two main diabetic traits (FBG and HbA1c) reached significant associations with* AGT* genotypes. We earlier indicate that these associations were independently associated. This strongly suggests that a change in FBG and HbA1c levels attributable to* AGT* genotypes has a metabolic impact on the risk of T2DM. With reference to* AGT* rs699, patients carrying the C-allele exhibit a trend of significant reduction in FBG and HbA1c levels. Our results also showed an association between haplotype TCG inferred from* AGT* variants with risk of T2DM, with no line of evidence points to significant gene-gene interactions. These findings support the key role of angiotensinogen in insulin resistance and genetic variants of* AGT* can serve as a predictive marker of T2DM. To explore this further, it may be informative to assess the angiotensinogen levels by genotype, which we did not investigate in this study. Current knowledge has suggested that AngII, the final product of RAS cascade, is working in concert with oxidative stress to induce insulin resistance [25]. Future genetic association studies of RAS should therefore examine both the AngII and oxidative stress levels in order to gain insight into this mechanism, although we note there is some evidence for association of* AGT* genotypes with reduced diabetic parameters.

The present study also identified two important factors that contributed to the susceptibility to T2DM, particularly male gender and waist circumference. Although we showed that the variants of the* AGT* were independently associated with T2DM, the influence of these factors in T2DM warrans further discussion. Asian men are at greater risk for T2DM compared to women [10]. Unlike the Caucasians who need to be relatively big to get diabetes, the Asians develop diabetes at even lower degree of obesity [26]. Similar trend is seen when comparing with the Europeans, where both BMI and waist circumference are lower in the South Asian men but the visceral adiposity is larger [27]. The notion that one's BMI measures one's waist and thus indicating diabetes, however, cannot be extrapolated at the Asian level. India for example, despite having low prevalence of obesity, they have notably high rates of T2DM [28]. Our data indicate that genetic variants of* AGT* in men make them more predisposed to T2DM and out of the 290 patients with T2DM, only 24% were obese. There is a likelihood of 53% risk reduction of T2DM in men subjected to variation in* AGT* rs699 and 47% risk reduction when confounded by waist circumference. In other word, the risk of diabetes is relatively high in Asian men with greater waist circumference, and the risk is higher when presented with susceptible genetic variations. The findings suggest that male gender and waist circumference are among the important factors need to be considered when analysing data involving Asian population, and this is one of the strengths in this study.

Results from studies of complex diseases like diabetes are not easily reproducible in different set of population commonly due to polygenic inheritance and gene-environment interactions [29]. In this study, we have eliminated the gene-gene interaction and confounding effects. Thus, our results fall within the assumption that the genetic variants of the* AGT* were independently associated with T2DM. Nevertheless, the gene-environment interactions such as physical activity and diet were not investigated and thus could be a limitation of this study. This limitation has been shared by many other genetic association studies including GWAS. More importantly, the GWAS has a limited success to identify new gene variants (especially those with low allele frequency) associated with disease susceptibility owing to the complexity of the disease and the key element of exposure and further substantiated by the number of independent tests performed [30]. Another limitation is associated with relatively small sample size of the patients. The said high prevalence of T2DM among the Asians should reflect a greater number of T2DM patients in our study; however, this was not achieved. This limitation is conditioned by several factors: (i) we recruited only Malay T2DM participants; (ii) the catchment area is more populated by the Chinese; and (iii) this study includes only a cohort from one centre. Measurement of plasma angiotensinogen levels would have been useful in view of the proposed importance of AngII, a breakdown product of angiotensinogen, in accentuating the T2DM risk in men, but these measurements have not been made. However, studies have demonstrated that angiotensinogen levels are strongly correlated with* AGT* variants [22]. Hence, further replication studies are required to confirm these findings.

## 5. Conclusion

Taken together, the present study provides a support on the association of genetic variants of the* AGT* gene with risk of T2DM. These RAS variants could serve as independent genetic predictors for the T2DM in the area of personalized medicine involving metabolic diseases. Further studies that include more variants of the RAS and examine the angiotensinogen and oxidative stress levels are necessary to dissect the potential role of these genetic variants as a predictive genetic marker for T2DM.

## Supplementary Material

Supplementary Table 1: Allele and genotype frequencies of SNPs and their association status with diabetes.
Supplementary Table 2: Association of SNPs with diabetes in the male gender.
Supplementary Table 3: Comparison of various clinical and biochemical parameters between the *AGT rs699* genotypes.

## Figures and Tables

**Table 1:** Demographic and clinical data of participants.

Characteristics	*n* (%) or mean ± SD		*P* value
	Control (*n* = 267)	DM (*n* = 290)	
Gender			0.824^a^
Male	108 (40)	120 (41)	
Female	159 (60)	170 (59)	
Age	57.0 ± 6.9	57.4 ± 6.2	0.618
BMI (kg/m^2^)	26.9 ± 4.9	27.3 ± 4.6	0.317^b^
Waist circumference (cm)	86.3 ± 12.7	91.8 ± 11.9	<0.000^b^
Waist-to-hip ratio	0.87 ± 0.1	0.90 ± 0.1	<0.0001
Systolic blood pressure (mmHg)	131.7 ± 15.5	133.7 ± 15.8	0.058
Diastolic blood pressure (mmHg)	78.8 ± 10.3	77.8 ± 8.5	0.650
Fasting blood glucose (mmol/L)	5.3 ± 0.7	8.4 ± 3.1	<0.0001
HbA1c (%) (mmol/mol)	5.5 ± 0.5 (36 ± 6)	8.8 ± 1.9 (73 ± 21)	<0.0001
Serum urea (mmol/L)	5.5 ± 7.8	5.3 ± 1.8	<0.0001
Serum creatinine (mmol/L)	76.9 ± 22.6	86.3 ± 25.3	<0.0001
Glomerular filtration rate (mL/min/1.7 m^2^)	85.4 ± 25.1	77.4 ± 23.3	0.988^b^
Triglyceride (mmol/L)	1.8 ± 5.2	1.8 ± 0.9	<0.0001
Total cholesterol (mmol/L)	5.2 ± 3.8	4.8 ± 3.2	<0.0001
HDL cholesterol (mmol/L)	1.9 ± 8.2	1.6 ± 5.9	<0.0001
LDL cholesterol (mmol/L)	3.0 ± 0.9	2.6 ± 1.0	<0.0001

^a^ *P* values were obtained using Pearson's *χ* ^2^ test, ^b^ *P* values were obtained using independent *t*-test, and other comparisons used Mann-Whitney *U* test.

**Table 2:** Demographic and clinical data of male participants.

Characteristics	Mean ± SD		*P* value
	Control (*n* = 108)	DM (*n* = 120)	
Age (years)	57.4 ± 6.8	58.13 ± 6.2	0.753^a^
BMI (kg/m^2^)	26.7 ± 4.1	27.2 ± 4.1	0.986^a^
Waist circumference (cm)	90.8 ± 11.1	95.3 ± 11.7	0.003^a^
Waist-to-hip ratio	0.90 ± 0.1	0.94 ± 0.1	<0.0001
Systolic pressure (mmHg)	131.30 ± 15.7	133.78 ± 14.9	0.112
Diastolic pressure (mmHg)	79.11 ± 10.3	78.22 ± 9.0	0.676
Fasting blood glucose (mmol/L)	5.29 ± 0.7	8.33 ± 2.8	<0.0001
HbA1c (%) (mmol/mol)	5.44 ± 0.5 (35.93 ± 5.6)	8.65 ± 2.0 (70.98 ± 21.7)	<0.0001
Serum urea (mmol/L)	5.28 ± 2.3	5.42 ± 1.6	0.101
Serum creatinine (mmol/L)	91.70 ± 19.5	99.60 ± 23.5	0.022
Glomerular filtration rate (mL/min/1.72 m^2^)	79.20 ± 19.2	75.40 ± 21.1	0.232^a^
Triglyceride (mmol/L)	2.35 ± 8.1	1.78 ± 1.0	0.276
Total cholesterol (mmol/L)	5.38 ± 5.9	4.50 ± 1.2	0.005
HDL cholesterol (mmol/L)	1.24 ± 0.4	1.98 ± 9.2	0.088
LDL cholesterol (mmol/L)	2.94 ± 0.9	2.53 ± 0.9	<0.0001

^a^ *P* values were obtained using independent *t*-test and other comparisons used Mann-Whitney *U* test.

**Table 3:** Association of AGT SNPs with diabetes in the male gender.

AGT	Allele frequency		Unadjusted *P* value	Adjusted *P* value^a^	OR^a^ (95% CI)
	Control	DM			
rs699 (T/C)					
T	0.12	0.21	1		Reference
C	0.88	0.79	0.017	0.007	0.47 (0.27–0.81)
TT			1		Reference
TC			0.815	0.803	0.80 (0.14–4.66)
CC			0.395	0.209	0.33 (0.60–1.85)
rs4762 (C/T)					
C	0.84	0.90	1		Reference
T	0.16	0.10	0.048	0.042	0.54 (0.29–0.98)
CC			1		Reference
CT			0.076	0.065	0.53 (0.27–1.04)
TT			0.245	0.320	0.31 (0.03–3.14)
rs5051 (A/G)					
A	0.88	0.80	1		Reference
G	0.12	0.20	0.032	0.017	1.94 (1.13–3.36)
AA			1		Reference
AG			0.022	0.026	2.09 (1.09–4.00)
GG			0.414	0.226	2.87 (0.52–15.75)

^a^Adjusted for waist circumference, age, and BMI.

**Table 4:** Association of haplotypes in AGT gene with diabetes in the male gender.

Haplotype	Control (%)	Case (%)	*P* value^a^	OR^b^ (95% CI)
CCA	0.721	0.691	0.457	0.859 (0.577–1.281)
TCG	0.117	0.202	0.012	1.917 (1.149–3.200)
CTA	0.157	0.103	0.078	0.615 (0.357–1.059)

^a^ *P* value was based on 100,000 permutations and ^b^OR was calculated using R program.
