# Supplementary material for: Renin-Angiotensin System Gene Variants and Type 2 Diabetes Mellitus: Influence of Angiotensinogen
Source: J Diabetes Res. 2015 Nov 22;2016:2161376. doi: 10.1155/2016/2161376 (PMC4670722; doi:10.1155/2016/2161376)
Supplement: Supplementary file 1 — Supplementary Table 1: Allele and genotype frequencies of SNPs and their association status with diabetes. Supplementary Table 2: Association of SNPs with diabetes in the male gender. Supplementary Table 3: Comparison of various clinical and biochemical parameters between the AGT rs699 genotypes. [file 2161376.f1.pdf]

Supplementary information

Supplementary Table 1: Allele and genotype frequencies of SNPs and their association status with diabetes

SNPs	Allele Frequency		Association	
	Control	DM	p-value	OR (95% CI)
ACE<br>Ins/Del	Ins = 0.74<br>Del = 0.26	Ins = 0.77<br>Del = 0.23	0.240	0.842 (0.642 - 1.104)
ACE<br>rs4459609 (A>C)	A = 0.74<br>C = 0.26	A = 0.76<br>C = 0.24	0.333	0.866 (0.661 - 1.136)
ACE<br>rs1800764 (T>C)	T = 0.72<br>C = 0.28	T = 0.77<br>C = 0.23	0.05	0.758 (0.578 - 0.994)
ACE<br>rs4344 (G>A)	G = 0.26<br>A = 0.74	G = 0.23<br>A = 0.77	0.369	1.143 (0.871 - 1.500)
ACE<br>rs4359 (C>T)	C = 0.71<br>T = 0.29	C = 0.76<br>T = 0.24	0.070	0.774 (0.593 - 1.011)
ACE<br>rs4363 (G>A)	G = 0.28<br>A = 0.72	G = 0.25<br>A = 0.75	0.204	1.199 (0.919 - 1.565)
AGT<br>rs699 (T>C)	T = 0.15<br>C = 0.85	T = 0.18<br>C = 0.82	0.127	0.771 (0.561 - 1.059)
AGT<br>rs4762 (C>T)	C = 0.87<br>T = 0.13	C = 0.88<br>T = 0.12	0.646	0.906 (0.637 - 1.289)
AGT<br>rs5051 (A>G)	A = 0.85<br>G = 0.15	A = 0.82<br>G = 0.18	0.196	1.249 (0.909 - 1.717)
AGTR1<br>rs388915 (T>C)	T = 0.86<br>C = 0.14	T = 0.84<br>C = 0.16	0.406	1.167 (0.838 - 1.626)

Supplementary Table 2: Association of SNPs with diabetes in the male gender

SNP	Allele Frequency		Unadjusted p-value	Adjusted p-value[a]	Adjusted OR[a] (95% CI)
	Control	DM			
ACE Ins/Del					
Ins	Ins = 0.75	Ins = 0.79	1		Reference
Del	Del = 0.25	Del = 0.21	0.289	0.583	0.877 (0.549 – 1.401)
Ins/Ins			1		Reference
Ins/Del			0.182	0.370	0.765 (0.425 – 1.375)
Del/Del			0.674	0.916	1.071 (0.302 – 3.798)
ACE rs4459609 (A>C)					
A	A = 0.75	A = 0.76	1		Reference
C	C = 0.25	C = 0.24	0.752	0.833	1.052 (0.659 – 1.678)
AA			1		Reference
AC			0.780	0.413	1.277 (0.711 – 2.293)
CC			0.332	0.566	0.687 (0.191 – 2.473)
ACE rs1800764 (T>C)					
T	T = 0.73	T = 0.78	1		Reference
C	C = 0.27	C = 0.22	0.280	0.565	0.871 (0.543 – 1.396)
TT			1		Reference
CT			0.954	0.698	1.121 (0.629 – 2.000)
CC			0.065	0.158	0.352 (0.082 – 1.501)
ACE rs4344 (G>A)					
G	G = 0.25	G = 0.21	0.289	0.451	1.196 (0.751 – 1.904)
A	A = 0.75	A = 0.79	1		Reference
GG			0.674	0.861	0.894 (0.256 – 3.126)
AG			0.182	0.343	0.753 (0.419 – 1.354)
*AA			1		Reference
ACE rs4359 (C>T)					
C	C = 0.71	C = 0.79	1		Reference
T	T = 0.29	T = 0.21	0.083	0.165	0.729 (0.467 – 1.139)
CC			1		Reference
CT			0.197	0.418	0.784 (0.434 – 1.414)
TT			0.141	0.189	0.468 (0.151 – 1.453)
ACE rs4363 (G>A)					
G	G = 0.28	G = 0.22	0.160	0.308	1.271 (0.802 – 2.017)
A	A = 0.72	A = 0.78	1		Reference
GG			0.440	0.638	0.745 (0.219 – 2.534)
AG			0.119	0.278	0.725 (0.405 – 1.297)
*AA			1		Reference
AGTR1 rs388915 (T>C)					
T	T = 0.85	T = 0.83	1		Reference
C	C = 0.15	C = 0.17	0.516	0.505	1.196 (0.707 – 2.023)
TT			1		Reference
CT			0.631	0.700	1.129 (0.609 – 2.095)
CC			0.465	0.504	1.829 (0.311 – 10.749)

[a]Adjusted for waist circumference in a multiple logistic regression model

*AA is used as reference because the frequency of A allele is higher

Supplementary Table 3: Comparison of various clinical and biochemical parameters between the *AGT* rs699 genotypes

Characteristics	AGT rs699, n=144 (Mean ± SD)			
	TT (n = 14)	CT (n = 158)	CC (n = 385)	P-value
Age (years)	57.6 ± 5.5	57.2 ± 6.4	57.2± 6.7	0.989
BMI (kg/m^2)	25.9 ± 6.5	27.3 ± 4.7	27.2 ± 4.8	0.593[a]
Waist circumference (cm)	85.3 ± 20.1	89.4 ± 11.5	89.4 ± 12.6	0.481
Waist-to-hip Ratio	0.9 ± 0.1	0.9 ± 0.1	0.9 ± 0.1	0.906
Systolic blood pressure (mmHg)	126.8 ± 9.9	135.0 ± 17.1	132.2 ± 15.6	0.103
Diastolic blood pressure (mmHg)	78.9 ± 6.5	77.8 ± 9.1	78.4 ± 9.6	0.766
Fasting blood glucose (mmol/l)	5.7 ± 1.2	7.5 ± 3.2	6.7 ± 2.5	0.005
HbA1c (%)	6.2 ± 1.0	7.6 ± 2.2	7.1 ± 2.3	0.012
Serum urea (mmol/L)	5.9 ± 2.5	6.4 ± 9.9	5.0 ± 1.8	0.107
Serum creatinine (mmol/L)	81.6 ± 17.4	83.5 ± 30.4	83.1 ± 26.2	0.982
Glomerular filtration rate (ml/min/1.72m^2)	82.2 ± 16.2	78.2 ± 26.2	81.6 ± 24.9	0.341[a]
Serum triglyceride (mmol/l)	1.3 ± 0.9	2.1 ± 6.6	1.7 ± 0.9	0.113
Serum cholesterol (mmol/l)	4.6 ± 1.2	5.1 ± 4.9	5.0 ± 2.7	0.505
High density lipoprotein (mmol/l)	1.3 ± 0.5	1.3 ± 0.4	1.9 ± 8.4	0.588
Low density lipoprotein (mmol/l)	2.7 ± 1.0	2.7 ± 1.1	2.8 ± 0.9	0.585

[a]P-values obtained using ANOVA, all other comparisons used Kruskal-Wallis test
